# Regression of Multiple Intracranial Meningiomas With Cessation of Progesterone Agonist Therapy: A Case Report

**DOI:** 10.7759/cureus.52479

**Published:** 2024-01-18

**Authors:** David S Bailey, Kevin John, Lekhaj C Daggubati, Brad E. Zacharia

**Affiliations:** 1 Department of Neurological Surgery, Penn State College of Medicine, Hershey, USA; 2 Department of Radiology, Stony Brook Medicine, Stony Brook, USA; 3 Department of Neurological Surgery, George Washington University Hospital, Washington, USA

**Keywords:** extra-axial lesion, hormonal influence on meningioma, megestrol acetate, progestin, meningioma

## Abstract

In this case report, we discuss a patient who experienced spontaneous regression of multiple intracranial meningiomas that were treated conservatively for 5 years after cessation of megestrol acetate, an exogenous progestin. In addition, we discuss the previous literature describing the relationship between exogenous progesterone medications and meningioma growth. This case, along with others reported, implies that cessation of progesterone therapy, when feasible, may alter the natural history of meningioma growth and thus impact treatment decisions.

## Introduction

Hormonal influence on meningioma growth has been reported for years, including in a patient described by Cushing, who experienced progression and subsequent regression of visual symptoms during and after pregnancy. This patient was later found to have a tuberculum sellae meningioma [1].

Since then, there have been several other case reports documenting similar alterations in the growth patterns of meningiomas during pregnancy. However, this anecdotal relationship has not been substantiated in epidemiological studies [2]. This natural phenomenon observed in pregnancy prompted studies that have demonstrated a relationship between exogenous hormonal supplements and meningioma growth. An association between meningioma growth and exogenous progesterone has been most definitively observed in progestin treatment for reproductive cancers. This association is also evident in hormone replacement therapy and the use of oral contraceptives [3]. While the exact physiology of this relationship is not fully understood, it may be explained by the common expression of estrogen and progesterone receptors in meningioma [4].

In this case report, we present a patient who experienced the spontaneous regression of multiple symptomatic meningiomas after cessation of megestrol acetate, a progesterone agonist used in the treatment of endometrial cancer. We present a description of our own case and conduct a literature review describing exogenous hormone supplementation and its potential impact on meningioma growth.

## Case presentation

**Figure 1:**
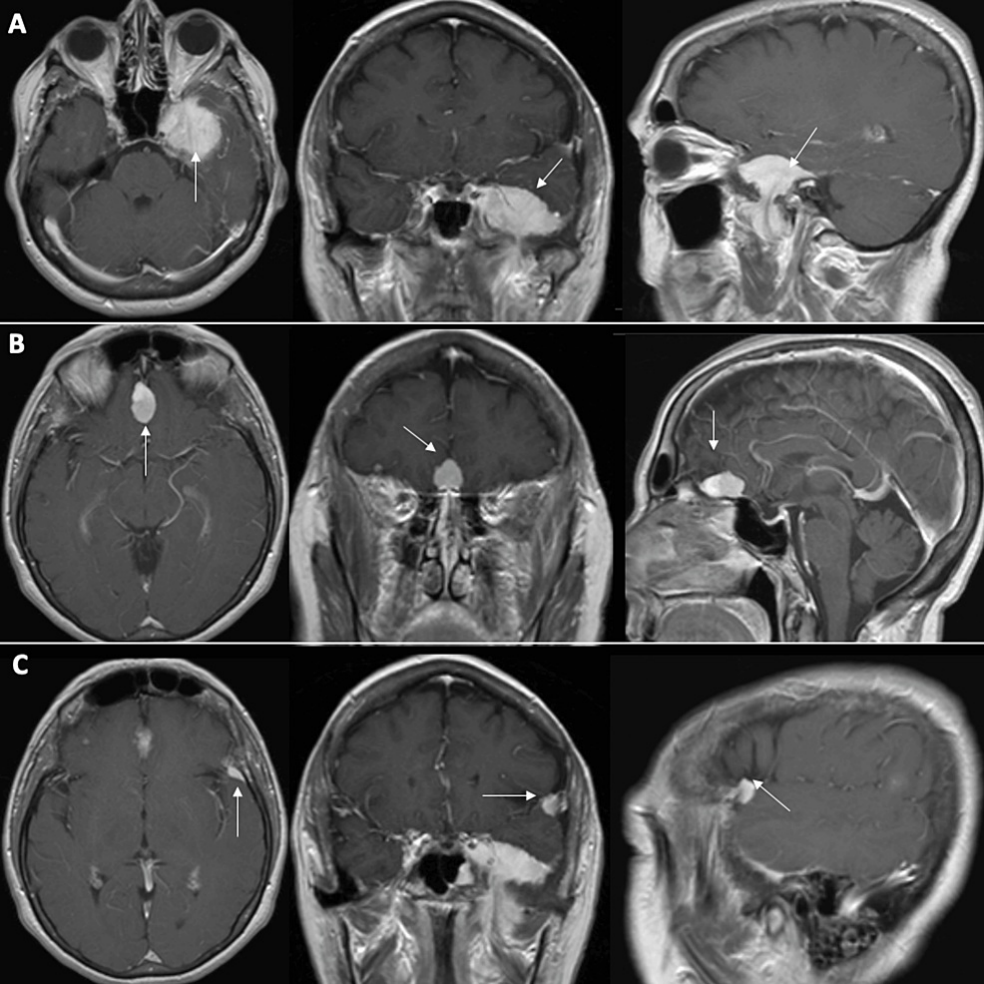
Initial T1 post-contrast MRI. Axial, coronal, and sagittal images demonstrating (A) left sphenoid meningioma (14.9 cc), (B) planum sphenoidale meningioma (1.9 cc), and (C) left lateral sphenoid wing meningioma (0.3 cc). MRI, magnetic resonance imaging

The patient was referred to our clinic in 2017 by her otolaryngologist following a computed tomography (CT) scan of the head, conducted to assess ear fullness. The scan revealed findings suggestive of multiple meningiomas. Magnetic resonance imaging (MRI) of the brain subsequently demonstrated three distinct extra-axial lesions consistent with meningioma. The largest of the three meningiomas measured 3.6 cm x 3.6 cm x 2.3 cm (14.9 cc) and was in the left sphenoid region. It exhibited invasion into the cavernous sinus, foramen ovale, and infratemporal fossa, likely leading to eustachian tube dysfunction. In addition, there was a planum sphenoidale meningioma measuring 2.3 cm x 1.3 cm x 1.3 cm (1.9 cc), along with a left-sided enhancing meningioma in the lateral sphenoid wing measuring 1.1 cm x 0.6 cm x 1.0 cm (0.3 cc) (Figure 1).

**Figure 2:**
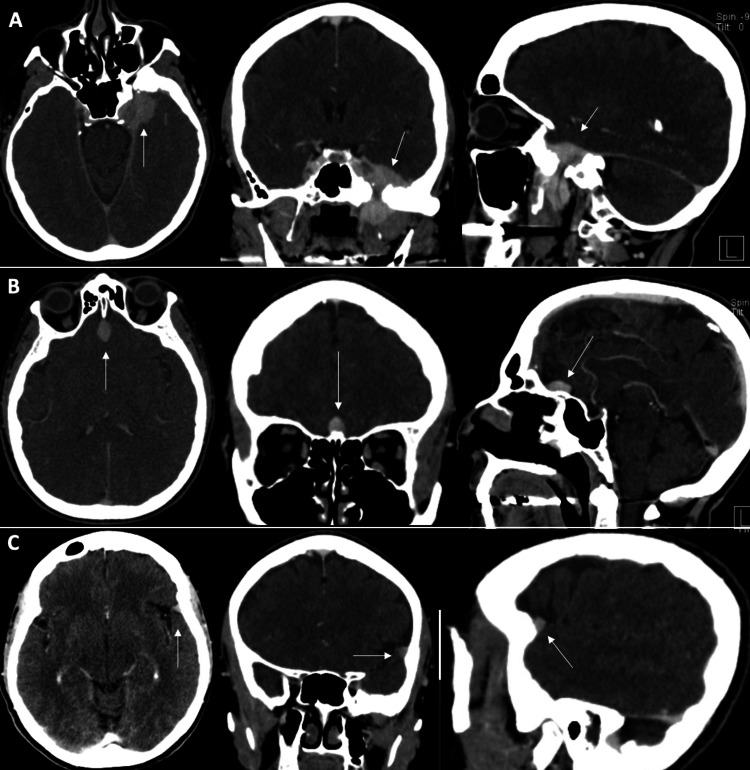
Two-year follow-up CT scan with contrast. Decreased size of (A) left sphenoid, (B) planum sphenoidale, and (C) left lateral sphenoid wing meningioma. CT, computed tomography

The patient had a past medical history of endometrial cancer, which was resected in 2003 and is currently in remission. Since then, she had been on maintenance therapy of 40 mg of megestrol acetate daily but had stopped taking it three weeks before her initial visit. We discussed observation versus surgical resection or radiosurgery for these lesions at her initial consultation. As her symptoms of ear fullness and subtle hearing loss were tolerable, we recommended an initial observation period with short-interval imaging. Follow-up imaging demonstrated gross stability of the lesions. At her two-year follow-up, a contrasted CT scan, conducted due to a gadolinium allergy, revealed a substantial decrease in size of the three meningiomas: left sphenoid region (2.4 cm x 2.6 cm x 1.6 cm, 5 cc), planum sphenoidale (1.5 cm x 1.1 cm x 0.8 cm, 0.7 cc), and left lateral sphenoid wing (0.9 cm x 0.5 cm x 0.8 cm, 0.2 cc) (Figure 2).

**Figure 3:**
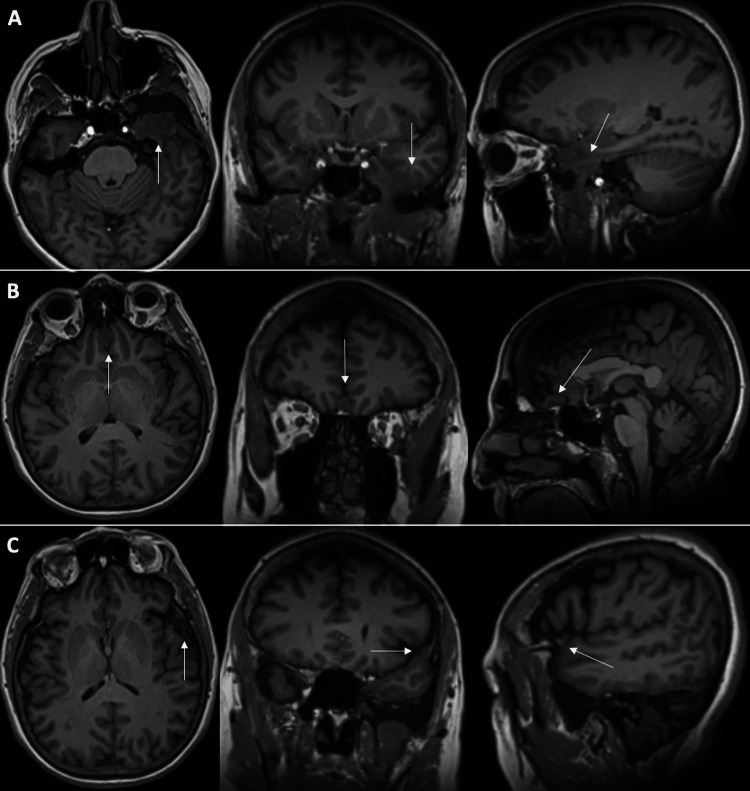
Five-year follow-up T1 non-contrasted MRI of the brain. Decreasing size of all three meningiomas: (A) left sphenoid, (B) planum sphenoidale, and (C) left lateral sphenoid wing. MRI, magnetic resonance imaging

The pattern continued at her most recent five-year follow-up, with a further decrease in size observed on a non-contrast brain MRI: Left sphenoid region (1.8 cm x 2.7 cm x 1.3 cm, 3.2 cc), planum sphenoidale (1.3 cm x 0.8 cm x 0.7 cm, 0.4 cc), and left lateral sphenoid wing (0.8 cm x 0.4 cm x 0.7 cm, 0.1 cc) (Figure 3).

This represented a total follow-up period of five years, spanning from 2017 to 2022. Throughout follow-up, the patient remained off megestrol acetate. During this time, she continued to do well and experienced a resolution of her ear fullness. She now reports being able to hear without deficit. In total, the patient has undergone a 79% volumetric decrease in her left sphenoid meningioma, a 21% decrease in the planum sphenoidale meningioma, and a 77% decrease in the left lateral sphenoid wing meningioma over the five years of follow-up (Table 1).

**Table 1:** Measurements over five-year follow-up.

Lesion	Presenting	Three-year follow-up	Five-year follow-up	Total percentage decrease
Left sphenoid	14.9 cc	5 cc	3.2 cc	79%
Planum sphenoidale	1.9 cc	0.7 cc	0.4 cc	21%
Left lateral sphenoid wing	0.3 cc	0.2 cc	0.1 cc	77%

Considering the progressive decrease in the size of her lesions, we intend to continue observation and will schedule her next follow-up appointment in two years.

## Discussion

Previous research has demonstrated a dose-response relationship between cyproterone acetate (CPA), a progestin medication that is used in Europe and Canada for the management of reproductive cancers, and meningioma growth. Initial anecdotal reports of CPA-associated meningiomas prompted epidemiologic studies that confirmed a dose-dependent risk of meningioma development with the use of this progestin medication [5-9]. These findings prompted regulatory restrictions on the use of CPA due to the risk for meningioma.

Similarly, the use of chlormadinone acetate (CMA) and nomegestrol acetate (NOMAC), both progestin medications with similar applications as CPA, have had anecdotal reports of meningioma progression with use and subsequent regression with cessation. This led to two recent database studies that revealed an elevated risk of meningioma associated with these agents, prompting regulatory action in France [3]. Less commonly reported are instances of megestrol acetate, another progestin medication with similar applications, affecting the growth pattern of intracranial meningioma. The first mention of megestrol acetate and meningioma comes from a study by Grunberg and Weiss who attempted to use megestrol acetate as a treatment for unresectable meningioma. There was no progression of their meningioma. Ultimately, the study was terminated after 12 months due to a lack of efficacy and significant systemic toxicity [10].

In addition to this therapeutic trial, there are two case reports in the literature describing growth variations in meningioma with the use of megestrol acetate. Vadivelu et al. reported a case of a patient on megestrol acetate for many years who was found to have multiple meningiomas. One of the lesions was resected, and pathology demonstrated a high expression of progesterone receptors. The authors discontinued megestrol acetate therapy and observed a decrease in the size of the other meningiomas over five years [11].

Similarly, Gruber et al. reported the management of four patients on megestrol acetate who were found to have intracranial meningioma. One patient was able to discontinue megestrol acetate after the initial surgical resection. The tumor recurred and was re-resected. The patient had 10 other tumors that were treated with a combination of stereotactic radiosurgery and open resection. After the initial recurrence, there was no further growth after nine years of follow-up. The other three patients had surgical resection of meningioma but were unable to cease megestrol acetate due to the progression of their underlying endometrial cancer diagnosis [12].

Our report is the third to describe a possible relationship between meningioma growth patterns and megestrol acetate. In our case, the patient experienced a decrease in the size of her multiple meningiomas with the cessation of megestrol acetate. This is similar to the early case reports of CPA, CMA, and NOMAC that resulted in more formal epidemiologic studies and subsequent regulatory action. While our report, along with those of Gruber et al. [12] and Vadivelu et al. [11], is anecdotal, the association between megestrol acetate and the growth patterns of intracranial meningiomas, coupled with existing knowledge about similar progestin analogs, implies that further research may be necessary to better elucidate this phenomenon on a larger scale.

More immediately, the findings presented here, along with the understanding of the relationship between meningioma growth patterns and exogenous progestin analogs, suggest that a more conservative approach may be feasible. If a patient presents with meningioma and is on a progestin medication that can be stopped, discontinuing the medication may be indicated, provided that the symptoms of the meningioma or the cessation of the medication are tolerable.

## Conclusions

This case adds to the existing evidence linking megestrol acetate to the growth patterns of intracranial meningiomas. While the current evidence is anecdotal, the consistent pattern across various similar progestin analogs underscores the importance of further investigation on a larger scale. Our findings advocate for a conservative approach to patients on megestrol acetate who are found to have meningioma. Discontinuing megestrol acetate when possible, especially if meningioma-related symptoms remain tolerable, can be a valid first step in the management of this specific patient population.
